# Premalignant and Malignant Skin Lesions in Two Recipients of Vascularized Composite Tissue Allografts (Face, Hands)

**DOI:** 10.1155/2015/356459

**Published:** 2015-10-13

**Authors:** Jean Kanitakis, Palmina Petruzzo, Aram Gazarian, Sylvie Testelin, Bernard Devauchelle, Lionel Badet, Jean-Michel Dubernard, Emmanuel Morelon

**Affiliations:** ^1^Department of Dermatology, Edouard Herriot Hospital Group, 69003 Lyon, France; ^2^Department of Surgery, University of Cagliari, 09124 Cagliari, Italy; ^3^Hand Surgery Department, Clinique du Parc, 69006 Lyon, France; ^4^Department of Maxillofacial Surgery and Stomatology, University Hospital Nord, 80054 Amiens, France; ^5^Department of Transplantation, Edouard Herriot Hospital Group, 69003 Lyon, France

## Abstract

Recipients of solid organ transplants (RSOT) have a highly increased risk for developing cutaneous premalignant and malignant lesions, favored by the lifelong immunosuppression. Vascularized composite tissue allografts (VCA) have been introduced recently, and relevant data are sparse. Two patients with skin cancers (one with basal cell carcinoma and one with squamous cell carcinomas) have been so far reported in this patient group. Since 2000 we have been following 9 recipients of VCA (3 face, 6 bilateral hands) for the development of rejection and complications of the immunosuppressive treatment. Among the 9 patients, one face-grafted recipient was diagnosed with nodular-pigmented basal cell carcinoma of her own facial skin 6 years after graft, and one patient with double hand allografts developed disseminated superficial actinic porokeratosis, a potentially premalignant dermatosis, on her skin of the arm and legs. Similar to RSOT, recipients of VCA are prone to develop cutaneous premalignant and malignant lesions. Prevention should be applied through sun-protective measures, regular skin examination, and early treatment of premalignant lesions.

## 1. Introduction

Recipients of solid organ transplants (RSOT) are at increased risk for developing various types of cancer, favored by the life-long immunosuppressive treatment (IST) necessary to avoid graft rejection. Skin cancers are the most common ones [1]. The incidence of skin cancer in RSOT increases with time after graft and with age of the recipient. It varies in the United States and Western Europe from 5% to 10–27% to 40–60% at 2, 10, and 20 years, respectively [2–5], with even higher figures observed in Australia, where the 20-year incidence reaches 70–82%. The commonest skin cancers are keratinocytic neoplasms, also known as nonmelanoma skin cancers (NMSC); they include, namely, squamous cell carcinomas (SCC) and basal cell carcinomas (BCC), the incidence of which is increased 65–250-fold and 10–16-fold, respectively, compared with matched control populations. SCC very often arise from premalignant lesions, namely, actinic keratoses (considered by many as* in situ* SCC) and porokeratoses, an uncommon dermatosis that may, rarely, progress toward SCC. NMSC developing in RSOT are often multiple and thereby entail significant morbidity and nonnegligible mortality in this patient group. Vascularized (composite) tissue allografts (VCA) are a more-recently introduced type of allografts aiming to replace nonvital tissues (such as face and hands), lost following severe trauma, or treatment of life-threatening or severely disfiguring tumours. VCA are immunogenic and necessitate lifelong antirejection IST, exposing their recipients to infectious and neoplastic complications of chronic immunosuppression. Up till now two VCA recipients (one hand, one face) have reportedly developed postgraft NMSC (BCC and SCC) [6, 7]. We report here two additional patients with VCA (face, hands) who developed premalignant and malignant skin lesions, including one BCC and one porokeratosis.

## 2. Case Reports

### 2.1. Patient 1

**Figure 1:**
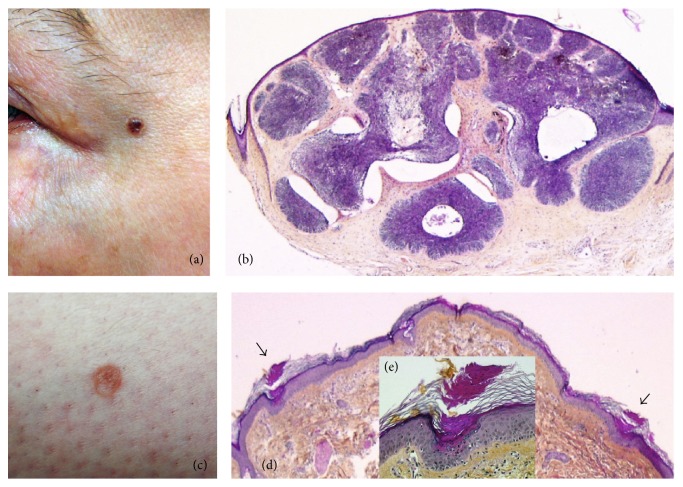
(a) Patient 1. Pigmented nodule on the left temple. (b) Histological examination shows a nodular-pigmented basal cell carcinoma, that is, a dermal proliferation of deeply basophilic nodules separated from the surrounding dermis by clefts. Melanin deposits (accounting for the clinically pigmented aspect of the tumour) are seen within the tumour (haematoxylin-eosin-saffron stain, ×100). (c) Patient 2. A 5 mm keratotic annular lesion of the leg. (d) Histological examination shows typical features of porokeratosis, that is, two vertical stacks of parakeratotic corneocytes (arrows) in the horny layer (cornoid lamella) corresponding to the edges of the lesion shown in (c). (e) (Inset) Cornoid lamella shown on the left of panel (d). A vertical stack of parakeratotic corneocytes is seated on a shallow depression of the epidermis; the latter is devoid of granular layer and contains apoptotic eosinophilic keratinocytes (haematoxylin-eosin-saffron stain, (d) ×100, (e) ×250).

The first facial transplantation was performed in 2005 by the Lyon and Amiens team, France. Details of the surgical procedure and the follow-up of this patient have been reported previously [8]. Briefly, the recipient was a 38-year-old woman (skin type II) severely bitten by her dog on May 2005. She received a partial face allograft on November 2005 from a brain-dead 46-year-old woman with whom she shared the same blood group and 5 HLA antigens. The induction IST included tacrolimus (Tac), mycophenolate mofetil (MMF), prednisone, and antithymocyte globulins; in addition, bone marrow cells of the donor were infused into the recipient on days 4 and 11 after transplantation. The maintenance IST consisted of sirolimus (SRL), MMF, and prednisone. SRL was introduced 11 months after transplantation because of increasing serum creatinine values. Extracorporeal photochemotherapy was also performed from August 2006 to December 2008. During the follow-up, the patient developed multiple mollusca contagiosa of the cheeks (both recipient and graft), HSV1 infection of the lips [8], and rosaceiform erythema of the graft likely due to repeated applications of local steroids [9], respectively, at five, seven, and 36 months after graft. Four years after transplantation the patient developed HPV+ cervical* in situ* carcinoma that was treated by conization. At the 6-year anniversary of follow-up (at the age of 44 years) the patient complained of a small asymptomatic, well-demarcated, pigmented nodule of the left temple close to the outer canthus that had appeared several months prior to consultation and was very slowly enlarging (Figure 1(a)). Clinically the lesion was suggestive of BCC and was surgically excised under local anaesthesia. Histological examination showed a typical aspect of nodular-pigmented BCC, that is, a dermal proliferation of deeply basophilic nodules separated from the surrounding dermis by clefts, containing focally melanin deposits (Figure 1(b)). Microscopic excision margins were clear and the tumour has not recurred after a 4-year follow-up. No additional (skin) tumours have been detected in this patient, who was reminded to adhere to sun-protective measures and to perform regular skin self-examination.

### 2.2. Patient 2

Details of this bilateral hand-allograft patient have been reported before [10]. Briefly, this skin type III patient suffered bilateral amputation at the midforearm level following electrocution on August 2004. She received a double hand allograft on February 2007 at the age of 27 years from a 40-year-female donor, with whom she had 4 HLA mismatches. Despite a maintenance IST based on steroids, MMF, and Tac, she developed during the following years several episodes of AR manifesting mostly as violaceous, occasionally scaly, papules over the dorsum of the hands and fingers. These were (temporarily) reversed with intravenous steroids, antithymocyte globulins, by increasing the oral steroid dose, or alemtuzumab. SRL was added to her IST in November 2010. In June 2015, that is, 8.4 years after graft, the patient reported the recent appearance of three small round annular keratotic lesions measuring 5–7 mm in diameter that had developed over the right arm and both shins (Figure 1(c)). Dermatoscopic examination showed the lesions to be sharply demarcated by a keratotic rim, suggestive of porokeratosis (PK). The diagnosis of disseminated superficial actinic PK was suggested clinically and confirmed by microscopic examination of the lesion of the right arm, after complete excision under local anaesthesia. Histological examination showed the presence of two vertical stacks of parakeratotic corneocytes within the orthokeratotic, basket-weave horny layer, realizing an aspect of cornoid lamella (Figure 1(d)). These were seated on a shallow depression of the underlying epidermis that was devoid of granular layer. The spinous layer contained dyskeratotic keratinocytes and the corresponding basal layer was vacuolated (Figure 1(e)). The lesions were treated by cryotherapy and the patient was reminded to perform regular skin self-examination and to refrain from sun-exposure.

## 3. Discussion

SOTR are at high risk to develop various skin premalignant and malignant lesions. Apart from the patients presented here, NMSC have been reported previously in two VCA recipients. The first patient was a 48-year-old woman with bilateral hand allotransplantation who developed BCC of the face 360 days after transplantation [6]. The second patient was a 54-year-old patient with face transplantation who developed two SCC (of the hand and the foot) during the first posttransplant year. This patient also developed a lymphoma; her graft was removed but the patient eventually died [7]. Of note, all these VCA recipients (including our two patients reported here) developed skin (pre)malignancies in their own skin and not that of the allograft. This may be simply due to the fact that the quantity of skin contained in the allografts is much lower than the recipient's own skin; therefore, admitting that both the patient's own skin and the allografted one have been exposed to the same load of carcinogenic factors (UV irradiation, IST), the former has a statistically higher risk than the latter to develop cutaneous premalignant and malignant lesions.

Posttransplant NMSC develop on average 8–10 years after grafting, and their numbers tend to increase with time [1]. Older RSOT are at higher risk, and this may explain why VCA recipients have been so far relatively spared, as they are on average younger than recipients of other organ transplants (kidney, heart, or liver). The risk of RSOT to develop BCC is increased 10–16-fold compared with the population at large, but the course of BCC is usually uneventful in RSOT provided they are completely excised [11–13]. In accordance with this, our first patient developed a slowly growing BCC that did not recur after complete excision, as was also the case in the other BCC previously reported in a hand-allograft recipient [6]. Regarding SCC, their incidence in SOTR is increased 65–250-fold, and these tumours may have an aggressive course, producing local recurrences and metastases that may prove fatal [14]. Therefore their early recognition and removal are of paramount importance. As SCC may develop from precursor lesions, namely, actinic keratoses, all such lesions should be treated early.

Our second patient developed PK, a rather uncommon skin disorder that may be seen in immunosuppressed patients, including RSOT. PK is thought to develop from the expansion of a clone of abnormal keratinocytes, favored by immunosuppression. The incidence of posttransplant PK varies according to the published series, from 0.34 to 10.7%, and the delay of appearance after graft in renal transplant patients is 4.5 years [15]. Although PK lesions are not malignant* per se*, they can transform into SCC, Bowen's disease (*in situ* carcinoma), or BCC [16] in 7–11% of cases; hence PK is often considered as a premalignant skin condition. In the setting of transplantation, PK cases progressing to malignancy are rare but may have an ominous course with metastases [17] and death [18]. Our second patient reported here is the first recipient of VCA to be diagnosed with this dermatosis. The clinical appearance (multiple disseminated lesions) was suggestive of the disseminated actinic superficial form (SDAPK). As its name implies, this specific form is triggered by UV-light, and this factor likely played a role (along with the IST) in our patient who had an important cumulative leisure sun-exposure (she was regularly practicing sailing prior to and after hand allotransplantation). Although SDAPK has a lower risk of progression to NMSC compared with other PK forms [15], we believe it should be treated appropriately in immunosuppressed patients.

## 4. Conclusion

Similar to other SOTR, VCA recipients may develop premalignant and malignant skin lesions. Their precise incidence in this patient group will be better assessed when a larger collection of cases will be available. Premalignant skin lesions and NMSC are favored by the IST that cannot be circumvented but are mainly due to sun-exposure; since this factor can be avoided, VCA recipients should be educated to protect themselves with sun-protective clothing and use of sunscreens. Regular skin self-examination, dermatological follow-up, and early detection and destruction of all premalignant lesions are strongly advisable, as in other RSOT. Secondary prevention also includes IST revision, for example, conversion to mTOR inhibitors that have been shown to decrease skin carcinogenesis in renal transplant recipients [19].

## Abbreviations

BCC: Basal cell carcinoma
DSAPK: Disseminated superficial actinic porokeratosis
HLA: Human leukocyte antigens
HPV: Human papillomavirus
IST: Immunosuppressive treatment
MMF: Mycophenolate mofetil
NMSC: Nonmelanoma skin cancer
PK: Porokeratosis
RSOT: Recipients of solid organ transplants
SCC: Squamous cell carcinoma
SRL: Sirolimus
Tac: Tacrolimus
VCA: Vascularized composite tissue allotransplantation.
